# Treatment of Acute Nonvariceal Upper Gastrointestinal Bleeding in Chinese Patients on Antithrombotic Therapy

**DOI:** 10.1155/2019/9190367

**Published:** 2019-12-18

**Authors:** Feng Gao, Xue Chen, Jie Zhang

**Affiliations:** Digestive Department, Beijing Anzhen Hospital, Capital Medical University, Beijing 100029, China

## Abstract

**Objective:**

To assess the treatment of acute nonvariceal upper gastrointestinal bleeding (ANVUGIB) in Chinese patients on antithrombotic therapy.

**Methods:**

The clinical data of patients with ANVUGIB who underwent upper gastrointestinal endoscopy 24 h after bleeding at Beijing Anzhen Hospital, Capital Medical University, from 2016 to 2018, were analyzed retrospectively. The patients were divided into antithrombotic therapy and control groups and into high-risk (Forrest Ia, Ib, IIa, and IIb) and low-risk (Forrest IIc and III) bleeding groups according to the results of endoscopy.

**Results:**

In all, 230 patients were enrolled, with 99 cases in the antithrombotic group (antiplatelet therapy 80 patients, anticoagulant therapy 19 patients) and 131 cases in the control group (without antithrombotic therapy). A total of 78 and 21 and 84 and 47 patients were at high- and low- risk for bleeding (*P* = 0.019) in the antithrombotic and control groups, respectively; 12.1% and 4.6% had esophageal bleeding (*P* = 0.047), and 8 and 2 patients received interventional therapy (*P* = 0.021). Overall, 21 patients with hemodynamic instability were treated via endoscopy with anesthesia under tracheal intubation and ventilator support: 20 patients in the antithrombotic group (13 patients within 1 month after coronary intervention, 5 patients within 1 month of cardiac-valve replacement, and 2 patients within 4 years of cardiac-valve replacement) and 1 patient with third-degree atrioventricular block in the control group. Ten patients received interventional therapy: eight and two in the two groups, respectively. Multidisciplinary consultation was conducted to regulate the use of antithrombotic drugs.

**Conclusion:**

Compared to the controls, patients in the antithrombotic group had a significantly higher incidence of critical and active bleeding. Patients with hemodynamic instability should be examined and treated via upper gastrointestinal endoscopy under anesthesia with tracheal intubation and ventilator support.

## 1. Introduction

The incidence of cardiovascular and cerebrovascular diseases has increased due to the aging of the population; therefore, an increasing number of patients will need antithrombotic therapy. Antithrombotic therapy reduces the incidence of thrombotic events but significantly increases the risk for bleeding. Bleeding is an important side effect of antithrombotic therapy, and antithrombotic therapy combined with bleeding significantly increases the risk of mortality. In addition, discontinuation of antithrombotic drugs after bleeding can significantly increase the risk of embolization [1–3]. Gastrointestinal bleeding may account for 54.9% of all bleeding events related to antithrombotic therapy [2]. However, there are no special Chinese consensus guidelines on how to perform endoscopy in antithrombotic patients with hemodynamic instability or when to discontinue or restore antithrombotic therapy after gastrointestinal bleeding, particularly in patients who need to continue antithrombotic therapy. Therefore, we retrospectively analyzed the clinical data of patients with acute nonvariceal upper gastrointestinal bleeding (ANVGIB) on antithrombotic therapy at Beijing Anzhen Hospital from 2016 and 2018 to provide reference information for the treatment of such patients.

## 2. Methods

### 2.1. Ethics

All methods and data analyses were approved by the local ethics board of Beijing Anzhen Hospital, Capital Medical University.

### 2.2. Subject Selection

Patients with gastrointestinal bleeding who were 18–84 years of age and treated at Beijing Anzhen Hospital, Capital Medical University, from 2016 to 2018 were enrolled retrospectively. The inclusion criteria were patients with ANVGIB (melena and/or hematemesis) who underwent emergency endoscopy within 24 h of bleeding and diagnosed with peptic ulcer. The exclusion criteria were detection of a tumor or variceal bleeding during emergency endoscopy, and bleeding at a location other than the upper gastrointestinal tract.

### 2.3. Data Collection

Patients' demographic and clinical data were collected, including age, sex, disease history, any antithrombotic drugs taken, results of upper gastrointestinal endoscopy, interventional treatments (and outcomes), discontinuation and restoration of antithrombotic therapy, and any hemodynamic instability (tachycardia or hypotension; defined as a heart rate of >120 beats/min, systolic blood pressure of <90 mmHg or a reduction in the systolic blood pressure from baseline of >30 mmHg, or a hemoglobin level of <50 g/L) [4, 5].

### 2.4. Patient Groups

**Figure 1:**
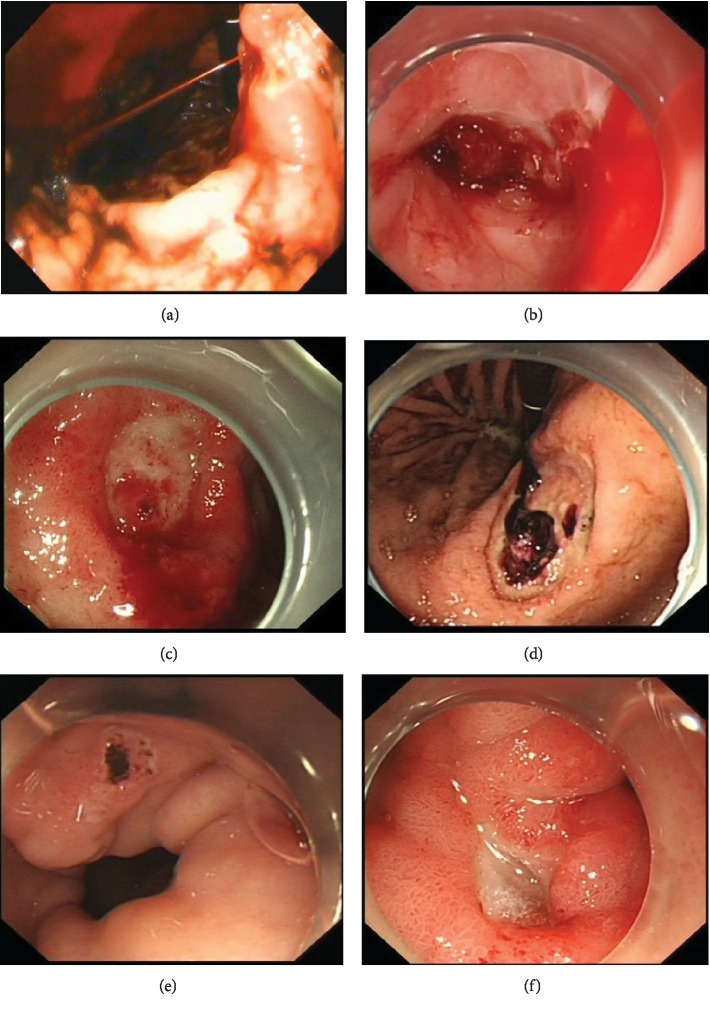
Forrest classification: (a) Ia spurting bleeding; (b) Ib oozing bleeding; (c) IIa nonbleeding visible vessel; (d) IIb an adherent clot; (e) IIc flat pigmented spot; and (f) III clean base ulcer.

The patients were divided into those that did and did not receive antithrombotic therapy (antithrombotic and control groups, respectively). They were subdivided into high-risk (Forrest Ia, Ib, IIa, and IIb) and low-risk (Forrest IIc and III) bleeding groups according to the Forrest classification [4–6] as follows: Ia spurting bleeding, Ib oozing bleeding, IIa nonbleeding visible vessel, IIb an adherent clot, IIc flat pigmented spot, and III clean base ulcer (Figure 1). The sites of bleeding were classified as the esophagus, stomach, and duodenum according to the results of the upper gastrointestinal endoscopy.

### 2.5. Endoscopic and Interventional Hemostatic Methods

Endoscopic hemostatic methods included local injection of drugs (epinephrine injection therapy), mechanical hemostasis (using through-the-scope clips), and local spraying of drugs (local spraying of thrombin and endoscopic spray type of medical glue) [4, 5]. For those who still had active bleeding, emergency interventional therapy (abdominal aorta angiography, celiac trunk angiography, and gastroduodenal artery or left gastric artery embolization) was performed.

### 2.6. Statistical Analyses

Categorical data were compared using chi-squared or Fisher's exact tests and are presented as numbers. Nonnormally distributed continuous data were compared using a Mann–Whitney *U* test and are presented as medians (interquartile range (IQR)). A value of *P* < 0.05 was considered indicative of statistical significance. Statistical analyses were performed using SPSS version 22.0 (IBM Corp., Armonk, NY).

## 3. Results

### 3.1. Demographic and Clinical Data

In all, 230 patients were enrolled: 99 in the antithrombotic group (antiplatelet therapy 80 patients, anticoagulant therapy 19 patients) and 131 in the control group. The sex distribution of the two groups was similar, while the mean age of patients was significantly older in the antithrombotic group than in the control group, and the proportion of patients on proton pump inhibitor (PPI) was significantly higher in the antithrombotic group than in the control group prior the bleeding occurring (Table 1).

Overall, 78 and 21 and 84 and 47 patients were at high and low risk for bleeding (*P* = 0.019) in the antithrombotic and control groups, respectively; 12.1% and 4.6% had esophageal bleeding (*P* = 0.047), and 8 and 2 patients received interventional therapy (*P* = 0.021).

A total of 21 patients with hemodynamic instability (which persisted despite ongoing volume resuscitation and red blood cell transfusion) were treated via endoscopy with anesthesia under tracheal intubation and ventilator support: 20 patients in the antithrombotic group (13 patients within 1 month after coronary intervention, 5 patients within 1 month of cardiac-valve replacement, and 2 patients within 4 years of cardiac-valve replacement) and 1 patient with third-degree atrioventricular block in the control group. Ten patients received interventional therapy: eight and two in the two groups, respectively.

### 3.2. Discontinue or Restart Antithrombotic Therapy after Gastrointestinal Bleeding

Antithrombotic therapy comprises antiplatelet therapy (APT) and anticoagulant therapy (ACT). APT in patients after acute myocardial infarction, coronary stent implantation, or coronary artery bypass graft includes one or two antiplatelet agents, typically enteric-coated aspirin with clopidogrel or tigrillo, with or without low-molecular-weight heparin (LMWH). ACT in patients with atrial fibrillation and/or cardiac valve replacement includes warfarin, rivaroxaban, and dabigatran. Patients in the low-risk bleeding group continued the original antithrombotic therapy without interruption. In patients in the high-risk bleeding group, if accompanying with the high risk for embolization, PCI (bare metal coronary artery stents within 1 month of placement or drug-eluting coronary artery stents within 12 months of placement), a prosthetic metal heart valve in the mitral position or prosthetic heart valve and atrial fibrillation or atrial fibrillation and mitral stenosis or <3 months after venous thromboembolism, continued antithrombotic therapy with an antiplatelet agent (clopidogrel or aspirin) or LMWH. When bleeding was stable for 3 to 7 days, the antithrombotic therapy was resumed. These patients underwent individualized antithrombotic therapy after multidisciplinary consultation and assessment of the risk for bleeding and embolization to increase the success rate of treatment and reduce the incidence of adverse events.

### 3.3. Prognosis

All patients achieved hemostasis after multidisciplinary consultation. In all, 228 patients were cured and discharged, and two died of heart failure and infection after cardiac valve replacement.

## 4. Discussion

At present, the Chinese consensus guideline on acute nonvariceal upper gastrointestinal bleeding mainly covers the diagnosis and treatment of gastrointestinal bleeding. However, there is no special Chinese consensus guideline on acute nonvariceal upper gastrointestinal bleeding in patients with antithrombotic therapy. Our hospital is one of the famous Chinese hospitals characterized by diagnosis and treatment of cardiovascular diseases; most of the patients are treated with antithrombotic therapy. Therefore, we have the opportunity to study these populations and provide some reference information for the treatment of such patients.

Most guidelines recommend ANVGIB patients to undergo early (≤24 h) emergency upper gastrointestinal endoscopy. For high-risk patients (i.e., those with hemodynamic instability, inpatients on hematemesis/nasogastric tube aspiration, and those with contraindications for discontinuation of antithrombotic therapy), very early (<12 h) emergency upper gastrointestinal endoscopy should be considered [5, 7]. A recently published large cohort study of 12,601 patients with peptic ulcer bleeding, of whom 44.6% were taking aspirin and 19.3% taking clopidogrel or anticoagulant drugs, showed that in patients with hemodynamic instability, emergency upper gastrointestinal endoscopy (6–24 h after admission) was associated with a lower in-hospital mortality rate [8]. Similarly, we performed upper gastrointestinal endoscopy within 24 h in 230 patients, 99 (43.0%) of whom were on antithrombotic therapy, 80 (34.8%) on APT, and 19 (8.2%) on ACT. Among the 99 patients on antithrombotic therapy, 78 (78.8%) were at high risk for bleeding (Forrest Ia, Ib, IIa, and IIb), a significantly higher proportion than in patients not on antithrombotic therapy (84/131, 64.1%). In all, 21 patients with hemodynamic instability (which persisted despite ongoing volume resuscitation and red blood cell transfusion) underwent endoscopy under anesthesia with tracheal intubation (20 in the antithrombotic group and 1 in the control group), 10 of whom (8 in the antithrombotic group and 2 in the control group, *P* = 0.021) received interventional therapy (abdominal aorta angiography, celiac trunk angiography, and gastroduodenal artery or left gastric artery embolization). Thus, more patients in the antithrombotic group suffered more severe illness and required endoscopic hemostasis combining with interventional therapy to stop bleeding and reduce the mortality rate. And study showed that rebleeding rate in the antithrombotic group is significantly higher than that in the control group (13.9% versus 5.8%, *P* = 0.02) than in the controls [9].

Tracheal intubation and ventilator support can maintain an open respiratory tract and prevent hypoxemia and aspiration caused by gastrointestinal bleeding, particularly in patients with acute upper gastrointestinal bleeding after cardiac surgery or those taking antithrombotic agents. The rate of esophageal bleeding was significantly higher in the antithrombotic group (12/99, 12.1% versus 6/131, 4.6%) for Bezold-Jarisch reflection (induced by hypovolemia and myocardial ischemia during or after cardiac surgery and percutaneous coronary intervention) [10, 11]. In addition, antithrombotic drugs reportedly induce esophageal ulcers (taking antithrombotic drugs in the recumbent position and/or insufficient water consumption, leading to drug retention and damage to the esophagus) [12–14].

In accordance with the guidelines for the diagnosis and treatment of ANVGIB [5, 7, 15], we administered a PPI bolus intravenously and then in continuous infusion (80 mg, then 8 mg/hour) for 72 h, followed by endoscopic local injection of a hemostatic agent (1 × 10,000 epinephrine saline), mechanical hemostasis (hemostatic clip), and endoscopic local application of a thrombin spray. For patients with persistent active bleeding, emergency interventional therapy (abdominal aorta angiography, celiac trunk angiography, and gastroduodenal artery or left gastric artery embolization) was performed. We have found that endoscopic hemostatic clips enable localization of the bleeding sites and reduce the duration of selective angiography.

**Figure 2:**
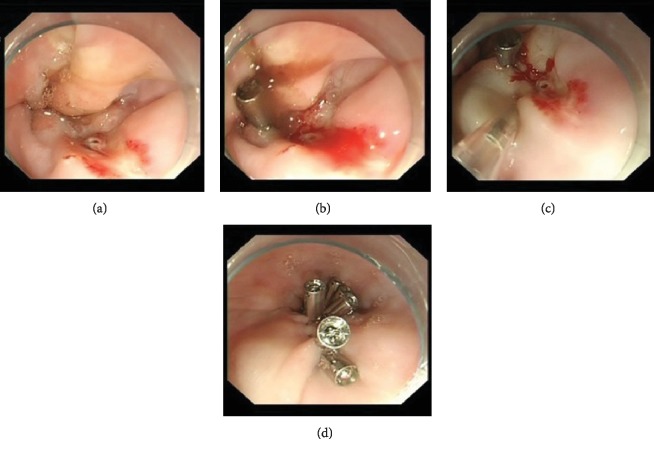
Endoscopic therapy of a lower esophageal ulcer with a nonbleeding visible vessel (Forrest IIa). (a) Lower esophageal ulcer with a nonbleeding visible vessel; (b) placement of a hemostatic clip to close the ulcer; (c) endoscopic local injection of a hemostatic agent (1 × 10,000 epinephrine saline) to constrict the vessel; and (d) placement of six hemostatic clips to close the ulcer.

The 2017 European Society of Cardiology (ESC) and the European Association for Cardio-Thoracic Surgery (EACTS) guidelines [16] recommend patients with severe bleeding (reduction in HGB of >5 g/dL) and hemodynamic instability to stop receiving dual antiplatelet therapy (DAPT) and commence single antiplatelet therapy (SAPT), preferably with a P2Y_12_ inhibitor. If bleeding persists despite treatment or treatment is not possible, all antithrombotic agents should be stopped and the need for DAPT or SAPT should be reevaluated, particularly for cases of upper gastrointestinal bleeding. If DAPT is restarted, a reduced treatment duration or switching to a less potent P2Y_12_ inhibitor (clopidogrel) should be considered, particularly in cases of recurrent bleeding. A 67-year-old male patient, diagnosed with unstable angina pectoris underwent PCI and bare-metal stent placement in March 2017, was prescribed enteric-coated aspirin (100 mg per day) plus clopidogrel (75 mg per day) and suffered hematemesis on April 19, 2017, when his HGB had decreased from 166 to 86 g/L. He underwent emergency endoscopy under anesthesia with tracheal intubation and ventilator support within 24 h, during which we identified a lower esophageal ulcer with a nonbleeding visible vessel (Forrest IIa, Figure 2). The ulcer was treated by endoscopic local hemostatic drug injection (1 × 10,000 epinephrine saline) and mechanical hemostasis (hemostatic clips) to stop the bleeding, and the enteric-coated aspirin was discontinued but clopidogrel (75 mg per day) was continued. The patient also received continuous PPI infusion (8 mg/h) for 72 h followed by an oral PPI (pantoprazole 40 mg per day). After the bleeding had been stable for 7 days, enteric-coated aspirin (100 mg per day) was restarted.

The European Society of Gastrointestinal Endoscopy (ESGE) and the European Society of Cardiology Working Group on Thrombosis [5, 17, 18] guidelines recommend APT for secondary prophylaxis in patients with ANVGIB. The recommendations are as follows: low-dose aspirin should be continued in patients of Forrest classifications IIc and III, while low-dose aspirin should be restarted in patients with a Forrest classification of Ia, Ib, IIa, or IIb after bleeding has been stable for 3–7 days. In patients with a Forrest classification of IIc or III, DAPT should be continued without interruption, while in those with a Forrest classification of Ia, Ib, IIa, or IIb, low-dose aspirin should be continued without interruption and an early cardiology consultation to decide whether to restart/continue the second APT is recommended. Second-look endoscopy at the discretion of the endoscopist may be considered to ensure the safety of clopidogrel in addition to aspirin.

**Figure 3:**
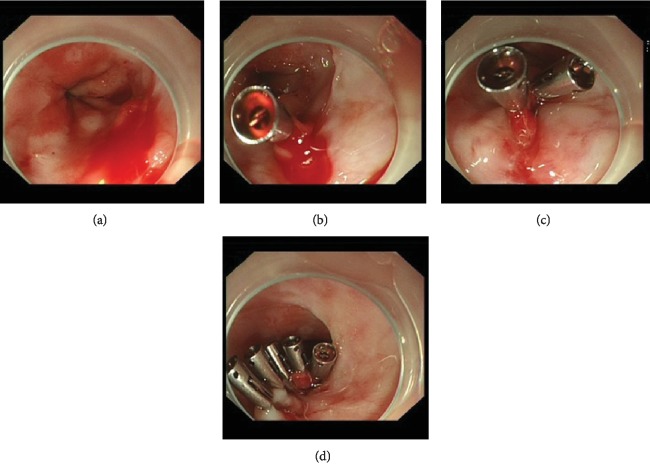
Endoscopic therapy of a lower esophageal ulcer with oozing bleeding (Forrest Ib). (a) Lower esophageal ulcer with oozing bleeding; (b) endoscopic local injection of a hemostatic agent (1 × 10,000 epinephrine saline) to constrict the vessel and placement of a hemostatic clip to close the ulcer; (c) placement of two hemostatic clips to close the ulcer; and (d) placement of five hemostatic clips to close the ulcer.

The current guidelines for patients on anticoagulation therapy [19–21] recommend restarting oral warfarin 7–15 days after the bleeding has stabilized. For patients at high risk for thrombosis, early LMWH bridging should be considered and oral warfarin should be restarted after day 7. Patients who develop gastrointestinal bleeding while taking dabigatran, rivaroxaban, or high-dose edoxaban, particularly those aged ≥75 years, should consider switching to apixaban 5 mg twice daily (2.5 mg twice daily if two or more of the following are present: age > 80 years, weight < 60 kg, and serum levels of creatinine > 133 *μ*mol/L). A PPI should also be prescribed, and *Helicobacter pylori* eradication therapy should be considered. If the patient is at high risk for gastrointestinal bleeding, switching to warfarin and strict INR control are recommended. A 71-year-old male patient who underwent mitral valve replacement in 2013 and was taking warfarin (4.5 mg per day) suffered hematemesis on June 7, 2018, when he had an HGB of 64 g/L and an INR of 4.68. The patient underwent emergency endoscopy under anesthesia with tracheal intubation and ventilator support within 24 h, during which a lower esophageal ulcer with oozing bleeding (Forrest Ib, Figure 3) was identified. The patient received endoscopic local injection of a hemostatic agent (1 × 10,000 epinephrine saline) and mechanical hemostasis (hemostatic clips) to stop the bleeding. He underwent LMWH bridging and restarted oral warfarin after the bleeding had been stable for 7 days.

In summary, compared to patients with ANVGIB not on antithrombotic therapy, those on antithrombotic therapy had a significantly higher incidence of critical and active bleeding. Patients with hemodynamic instability should be examined and treated by upper gastrointestinal endoscopy under anesthesia with tracheal intubation and ventilator support. Individualized antithrombotic therapy should be formulated by a multidisciplinary team based on assessment of the risk for bleeding and embolization to increase the success rate of treatment and reduce the incidence of adverse events.

## Figures and Tables

**Table 1:** Demographic and clinical data of patients with upper gastrointestinal bleeding.

Item	Antithrombotic group	Control group	Chi-squared or Fisher's exact test or Mann–Whitney *U* test	
	*n* = 99	*n* = 131	*P* value	
Age (median [IQR], y)	67.0 (18.0)	58.0 (30.0)	*Z* = −6.397	*P* < 0.001
Sex, M/F (*n*)	71/28	105/26	*χ* ^2^ = 2.233	*P* = 0.135
Forrest classification, high risk/low risk (*n*)	78/21	84/47	*χ* ^2^ = 5.824	*P* = 0.019
Bleeding sites, E/S/D (*n*)	12/26/61	6/52/73	*χ* ^2^ = 7.433	*P* = 0.024
Esophageal bleeding (*n*, %)	12 (12.1)	6 (4.6)	*χ* ^2^ = 4.445	*P* = 0.047
Endoscopy with anesthesia under tracheal intubation (*n*, %)	20 (20.2)	1 (0.7)	*χ* ^2^ = 25.680	*P* < 0.001
PPI therapy prior the bleeding (*n*, %)	43 (43.4)	11 (8.4)	*χ* ^2^ = 38.529	*P* < 0.001
Interventional therapy (*n*, %)	8/90	2/129	*χ* ^2^ = 5.913	*P* = 0.021
APT/ACT (*n*)	80/19	—	—	—
PCI or CABG ≤ 1 m/>1 m (*n*)	16/64	—	—	—
Cardiac valve replacement ≤ 1 m/>1 m (*n*)	5/14	—	—	—

IQR: interquartile range; E: esophagus; S: stomach; D: duodenum; APT: antiplatelet therapy; ACT: anticoagulant therapy; PCI: percutaneous coronary intervention; CAB: coronary artery bypass graft.

## Data Availability

The raw data used to support the findings of this study are available from the corresponding author upon request.
